# Less intensive lipid-lowering therapy after ST-elevation myocardial infarction is associated with cardiovascular events: 2-year follow-up of “Jena auf Ziel”

**DOI:** 10.1007/s00392-025-02736-y

**Published:** 2025-08-27

**Authors:** Franz Haertel, Umidakhon Makhmudova, Jens-Arndt Geiling, Bernward Lauer, Sven Möbius-Winkler, Sylvia Otto, P. Christian Schulze, Oliver Weingärtner

**Affiliations:** 1 Department of Internal Medicine I, Division of Cardiology, Angiology and Intensive Medical Care, Friedrich-Schiller-University, University Hospital Jena, Am Klinikum 1, Jena, Germany; 2 Deutsches Herzzentrum der Charité, Department of Cardiology, Angiology and Intensive Care Medicine, Berlin, Germany

**Keywords:** LDL-C, Lipid-lowering therapy, STEMI, Lipid clinic, Jena auf Ziel

## Abstract

**Background:**

“Jena auf Ziel” (“JaZ”) is a prospective cohort study in patients with ST-elevation myocardial infarction (STEMI). Early combination of a statin and ezetimibe was initiated on the day of admission and lipid-lowering therapy (LLT) was escalated during follow-up with bempedoic acid (BA) and PCSK9 inhibitors (PCSK9-I) to reach guideline-recommended LDL-cholesterol (LDL-C) levels. During the initial follow-up period of 12 months, all patients reached the recommended ESC/EAS LDL-C target for very high-risk patients of < 55 mg/dL.

**Methods:**

Twelve months after the index event, patients enrolled in “JaZ” had the option of either continuing with regular follow-ups in the outpatient lipid clinic of the university hospital Jena or transitioning to standard care by their general practitioners (GPs). Fifty-three patients (62%) stayed with the outpatient lipid clinic and 32 (38%) preferred treatment by their local GP. After 24 months, we analyzed differences in prescribed lipid-lowering drugs, LDL-C target attainment, LDL-C time on target, and major adverse cardiac events (MACEs = nonfatal ischemic cardiovascular events, admission for heart failure, nonfatal stroke) between groups.

**Results:**

All 85 patients enrolled in the initial study were followed up for 24 months. The average LDL-C after 24 months was 1.47 ± 0.71 mmol/L in the total study population. Fifty-one patients (60%) of the entire cohort were still on LDL-C target of 1.4 mmol/L or below (outpatient lipid clinic group: 72.5% vs. GP group: 27.5%; *p* = 0.037). The average LDL-C in patients followed up in the outpatient lipid clinic was significantly lower compared to patients who were treated by GPs (1.2 ± 0.7 mmol/L vs. 2.1 ± 1.04 mmol/L; *p* < 0.01). Moreover, patients in the outpatient lipid clinic had a longer time on LDL-C targets compared to patients treated by GPs (82.4 ± 29.5% vs. 62.4 ± 36.6%; *p* < 0.01). The main cause of missed LDL-C targets was deprescribing of LLT by local GPs, surpassing non-adherence (2.1 ± 1.04 mmol/L vs. LDL-C: 1.52 ± 0.53 mmol/L; *p* < 0.01). Patients with MACE during follow-up were characterized by a shorter time on LDL-C targets compared to patients without MACE (58.1 ± 29.9% vs. 79.1 ± 28.1%; *p* = 0.048) and higher LDL-C levels at 24 months (2.04 ± 1.26 mmol/L vs. 1.27 ± 0.72 mmol/L; *p* < 0.01).

**Conclusion:**

In this cohort of STEMI patients, a less intensive lipid-lowering strategy during a 2-year follow-up was associated with higher LDL-C levels and a higher incidence of MACE. Therefore, a regular follow-up in a specialized lipid outpatient clinic was superior to standard care treatment by general practitioners.

**Graphical abstract:**

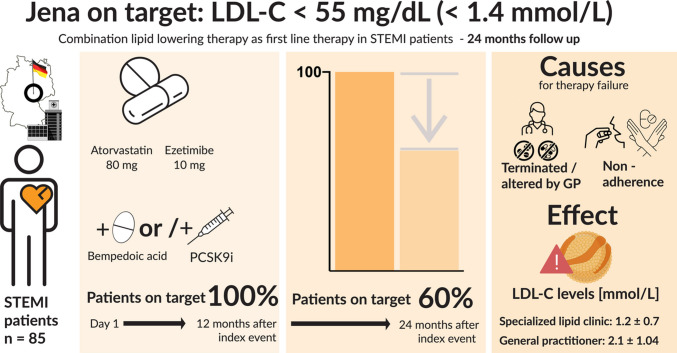

## Introduction

Cardiovascular disease (CVD) remains the leading cause of death in Germany, accounting for approximately 34% of all deaths in 2022 [1]. Despite a high-standard healthcare system, Germany lags behind other high-income countries in terms of cardiovascular outcomes and life expectancy due to shortcomings in prevention [2]. Elevated low-density lipoprotein cholesterol (LDL-C) levels stand as a pivotal modifiable risk factor for atherosclerotic cardiovascular disease (ASCVD) and a causal contributor to plaque development and adverse cardiovascular events [3, 4].

The 2019 European Society of Cardiology (ESC) and European Atherosclerosis Society (EAS) guidelines recommend a more aggressive LDL-C reduction for very high-risk patients recommending a more than 50% reduction from baseline and a target of < 55 mg/dL (< 1.4 mmol/L) [5]. The stepwise approach starts with a high-intensity statin, followed by ezetimibe and escalates to PCSK9 inhibitors (PCSK9-I) if targets are not achieved [5]. Only recently, a simulation study has demonstrated that more than 90% of patients could attain these targets with currently available lipid-lowering therapies (LLT) [6]. However, data from real-world registries such as SWEDEHEART, GOULD, SANTORINI, and DA VINCI consistently show that fewer than 20–25% of high-risk patients reach LDL-C goals in routine clinical practice [7–10]

In “Jena auf Ziel” (JaZ), a prospective, single-center cohort study, our group demonstrated that early combination of a high-intensity statin and ezetimibe is feasible, effective, and safe [11]. All patients admitted with ST-elevation myocardial infarction (STEMI) to Jena University Hospital were initiated on atorvastatin 80 mg and ezetimibe 10 mg in the cath lab after the procedure of the index event [11]. If LDL-C targets were not reached during follow-up, therapy was escalated either with bempedoic acid (BA) or PCSK9-I [11]. Eighty percent of the patients achieved LDL-C targets on combination of a statin and ezetimibe after 4–6 weeks, and after escalation with BA or PCSK9-I all patients reached ESC/EAS LDL-C targets [11]. Data from the SWEDEHEART registry showed that in patients with a diagnosis of a myocardial infarction, early and sustained LDL-C lowering after the index event by a combination of a statin and ezetimibe reduces cardiovascular outcomes compared to delayed combination or statin monotherapy [12]. Since delayed use of combined lipid-lowering therapy with a statin and ezetimibe was associated with more cardiovascular outcomes, the authors suggested that future care pathways should be streamlined and include the “Jena auf Ziel” approach with early combination of a statin and ezetimibe as standard of care [12].

Despite this success, however, Germany faces systemic barriers to consistent LDL-C target achievement, stemming largely from divergent guideline practices between office-based cardiologists (OBCs) and general practitioners (GPs). While OBCs in Germany follow the ESC/EAS recommendations, GPs adhere to the Nationale Versorgungsleitlinie (NVL), which recommends a “fire-and-forget” strategy and a less intensive LDL-C lowering (e.g., LDL-C target < 70 mg/dL for very high-risk patients) [5, 13].

“LipidSnapshot,” a joint initiative of the German Cardiac Society (Deutsche Gesellschaft für Kardiologie; DGK), the German Society of Lipidology (Deutsche Gesellschaft für Lipidologie; DGFL), and the Association of Office-Based Cardiologists (Bund der niedergelassenen Kardiologen; BNK), evaluated real-world LDL-C target achievement in GP and cardiologist settings [14]. Data of patients with established atherosclerotic cardiovascular disease (ASCVD) analyzed in 2023 revealed that ESC/EAS LDL-C targets were reached only in 12.1% of patients treated by GPs, but in 27.4% of the patients treated by cardiologists [14].

The discrepancies observed in “LipidSnapshot” prompted the question of whether comparable LDL-C targets can be documented over another 12-month period of follow-up of the “Jena auf Ziel” (JaZ) cohort if patients were continuously treated by the outpatient lipid clinic of the University Hospital in Jena or by their local GPs. Therefore, 12 months after the index event, patients enrolled in “JaZ” had the option of either continuing with regular follow-ups in the outpatient lipid clinic or transitioning to standard care by their GPs. In the current analysis, we evaluate differences in LDL-C goal attainment, time on LDL-C target, and cardiovascular outcomes between patients followed in the outpatient lipid clinic of the University Hospital in Jena or by their local GP 24 months after ST-elevation myocardial infarction (STEMI).

## Materials and methods

### Study design

“Jena auf Ziel—JaZ” is a prospective cohort study that included all patients admitted with STEMI to the Jena University Hospital between January 1 and December 31, 2021. The study received approval from the Local Ethics Committee (5219–07/17). Upon admission, patients were initiated on a combined lipid-lowering therapy (LLT) consisting of a high-intensity statin (atorvastatin 80 mg) and the cholesterol absorption inhibitor ezetimibe (10 mg). During their hospital stay, patients were educated on cardiovascular risk reduction. LDL-C levels were measured throughout the entire hospital stay and the lipid profile was recorded on individual patient cards to empower patients to LDL-C goal attainment. The primary outcome was LDL-C target attainment (< 1.4 mmol/L or < 55 mg/dL) within 12 months after the index event. If EAS/ESC targets were not reached, LLT was escalated either with bempedoic acid or PCSK9-I (alirocumab or evolocumab).

After 12 months of follow-up, patients could decide to continue either with regular follow-ups in the outpatient lipid clinic or to get routine standard care by their GP. After 24 months, we analyzed differences between these two strategies in regard to changes in lipid-lowering medications, LDL-C target attainment, LDL-C time on target, and major adverse cardiac events (MACE = nonfatal ischemic cardiovascular events, admission for heart failure, nonfatal stroke).

### Data management

Demographic, clinical, procedural, and laboratory parameters, along with diagnostic work-up data, were initially collected and anonymously recorded using the SAP electronic patient management system (SAP, Walldorf, Germany). The system automatically provided regular updates on the patients’ treatment status, whether they continued care at the outpatient lipid clinic of the University Hospital Jena or with their general practitioners (GPs).

### Statistical analysis

Statistical analyses were performed using SPSS Statistics (version 27.0, SPSS Inc., IBM, Armonk, New York, USA) and the online calculator (www.graphpad.com) of GraphPad Prism (GraphPad Software, Inc., Boston, Massachusetts, USA). A *p*-value of less than 0.05 was considered statistically significant. Baseline parameters are descriptively presented as whole integers and percentages. Data normality was assessed using the Kolmogorov–Smirnov test, and appropriate statistical methods were applied accordingly. The frequency of nominally scaled parameters was compared using Pearson's chi-squared test if the cells had an expected frequency of 5 or greater. Fisher’s exact test was used for cell counts less than 5, particularly in 2 × 2 and 3 × 2 contingency tables. Normally distributed data are expressed as mean ± standard deviation (mean ± SD) and was analyzed using the *t*-test for independent samples or one-way analysis of variance for mean comparisons. Non-normally distributed data are reported as medians with interquartile ranges (median [IQR: 25th percentile–75th percentile]) and analyzed using the Mann–Whitney *U*-test. Diagrams were generated using SPSS Statistics.

## Results

### Study population and clinical characteristics

Eighty-five patients were included in the study (Table 1). Mean age was 68.8 ± 12.8 years. The majority of the cohort was male (85.9%, *n* = 73), and all patients had confirmed coronary artery disease (CAD). Over the 24-month follow-up period, four patients died due to non-cardiac causes.

**Table 1:** Baseline characteristics of the study population

	Total study population	LDL-C goal attained after 24 months	LDL-C goal not attained after 24 months	*p* Value*
	(*n* = 85)	(*n* = 51 (60%))	(*n* = 34 (40%))	
**Demographics**				
Age [years, mean ± SD]	68.8 ± 12.8	64.8 ± 11.7	66.4 ± 13.8	n.s
Male [*n* (%)]	73 (85.9)	45 (88.2)	28 (82.3)	n.s
Female [*n* (%)]	12 (14.1)	6 (11.8)	6 (17.6)	n.s
BMI [kg/m^2^, mean ± SD]	27.7 ± 4.6	27.3 ± 4.4	27.7 ± 4.1	n.s
Smokers [*n* (%)]	33 (38.8)	20 (39.2)	13 (38.2)	n.s
Systolic blood pressure [mmHg, mean ± SD]	145.6 ± 27.7	141.6 ± 29.5	146.6 ± 23.5	n.s
Diastolic blood pressure [mmHg, mean ± SD]	79.9 ± 17.2	77.4 ± 18.9	80.1 ± 12.8	n.s
CAD [*n* (%)]	85 (100)	51 (100)	34 (100)	n.s
1 Vessel CAD [*n* (%)]	36 (42.3)	22 (43.1)	14 (41.2)	
2 Vessel CAD [*n* (%)]	23 (27.1)	14 (27.5)	9 (26.5)	n.s
3 Vessel CAD [*n* (%)]	26 (30.6)	15 (29.4)	11 (32.3)	
Number of stents [median (IQR)]	1 (1–2)	1 (1–2)	1 (1–2)	n.s
**Biomarkers**				
Total cholesterol [mmol/L, mean ± SD]	4.6 ± 1.2	4.3 ± 1.5	4.4 ± 1.4	n.s
LDL-C [mmol/L, mean ± SD]	3.2 ± 1.2	3.1 ± 1.3	3.3 ± 1.1	n.s
HDL-C [mmol/L, mean ± SD]	1.2 ± 0.3	1.1 ± 0.3	1.2 ± 0.2	n.s
Triglycerides [mmol/L, mean ± SD]	1.3 ± 1.3	1.6 ± 1.5	1.1 ± 0.5	n.s
Lipoprotein (a) [nmol/L, median (IQR)]	21.0 (20.0–67.5)	19.9 (19.9–71.0)	23 (19.9–136)	n.s
HbA1C [%, mean ± SD]	5.9 ± 0.8	6.0 ± 0.8	5.8 ± 0.7	n.s
eGFR [mL/min, mean ± SD]	75.8 ± 23.5	73.5 ± 24.0	79.8 ± 17.4	n.s
**Echocardiographic parameters**				
LVEF [%, mean ± SD]	50.7 ± 10.5	51.6 ± 11.9	48.3 ± 9.8	n.s
**Comorbidities**				
Hypertension [*n* (%)]	54 (63.5)	31 (60.7)	23 (67.6)	n.s
CKD [*n* (%)]	52 (61.2)	32 (62.7)	20 (58.8)	n.s
PAD [*n* (%)]	9 (10.6)	5 (9.8)	4 (11.7)	n.s
Diabetes [*n* (%)]	13 (15.3)	8 (15.6)	5 (14.7)	n.s

*p* < 0.05 = statistically significant, *SD* standard deviation, *BMI* body mass index, *PAD* peripheral artery disease, *CKD* chronic kidney disease, *CAD* coronary artery disease, *n.s.* not significant, *IQR* interquartile range, *eGFR* estimated glomerular filtration rate, *LDL-C* low-density lipoprotein cholesterol, *HDL* high-density lipoprotein, *LVEF* left ventricular ejection fraction

*LDL-C goal attained after 24 months vs. LDL-C goal not attained after 24 months

### Lipid-lowering therapy and LDL-C goal attainment

At the end of the 24-month follow-up period, 96% of patients were on statins. Among these, 71.8% were receiving atorvastatin, with dosages of 80 mg (75.7%), 40 mg (20.9%), and 20 mg (3.4%). Additionally, 28.2% of patients were on rosuvastatin, with doses of 40 mg (40.9%), 20 mg (45.5%), 10 mg (4.5%), and 5 mg (9.1%).

Furthermore, 87.7% of patients received ezetimibe, 17.3% were treated with BA, and 6.2% were on a PCSK9-I. The mean LDL-C level in the overall study population after 24 months was 1.47 ± 0.71 mmol/L.

Fifty-one patients (60%) reached the LDL-C target of ≤ 1.4 mmol/L, while 34 patients (40%) did not attain recommended LDL-C levels. At baseline, patients from the outpatient lipid clinic and patient from GPs were similar regarding the lipid profiles (LDL-C: 3.2 ± 1.2 vs. 3.2 ± 0.9 mmol/L; *p* > 0.05). Patients who continued treatment in the outpatient lipid clinic were significantly more likely to achieve LDL-C goals compared to those managed by GPs (72.5% vs. 27.5%, *p* = 0.037). Moreover, LDL-C levels were significantly lower in patients managed by the outpatient lipid clinic compared to those treated by GPs (1.2 ± 0.7 mmol/L vs. 2.1 ± 1.04 mmol/L, *p* < 0.01) (Table 2).

**Table 2:** Characteristics of the study group regarding patients from outpatient lipid clinic vs. GP

	Outpatient lipid clinic	GP	*p* Value*
	(*n* = 53 (62%))	(*n* = 32 (38%))	
**Demographics—baseline**			
Age [years, mean ± SD]	63.7 ± 11.5	65.6 ± 15.3	n.s
Male [*n* (%)]	46 (86.8)	27 (84.4)	n.s
Female [*n* (%)]	7 (13.2)	5 (15.6)	n.s
BMI [kg/m^2^, mean ± SD]	27.8 ± 5.2	28.0 ± 4.6	n.s
Smokers [*n* (%)]	21 (39.6)	12 (37.5)	n.s
Systolic blood pressure [mmHg, mean ± SD]	140.4 ± 27.3	154.5 ± 26.5	**0.024**
Diastolic blood pressure [mmHg, mean ± SD]	78.5 ± 18.6	82.2 ± 14.5	n.s
CAD [*n* (%)]	53 (100)	32 (100)	n.s
1 Vessel CAD [*n* (%)]	23 (43.4)	13 (40.6)	
2 Vessel CAD [*n* (%)]	14 (26.4)	9 (28.1)	n.s
3 Vessel CAD [*n* (%)]	16 (30.2)	10 (31.3)	
Number of stents [median (IQR)]	1 (1–2)	1 (1–3)	n.s
**Biomarkers—baseline**			
Total cholesterol [mmol/L, mean ± SD]	4.3 ± 1.4	4.1 ± 1.1	n.s
LDL-C [mmol/L, mean ± SD]	3.2 ± 1.2	3.2 ± 0.9	n.s
HDL-C [mmol/L, mean ± SD]	1.2 ± 0.4	1.1 ± 0.3	n.s
Triglycerides [mmol/L, mean ± SD]	1.5 ± 1.6	1.2 ± 0.6	n.s
Lipoprotein (a) [nmol/L, median (IQR)]	19.9 (19.9–86.5)	24.0 (19.9–64.0)	n.s
HbA1C [%, mean ± SD]	6.0 ± 0.9	5.8 ± 0.6	n.s
eGFR [mL/min, mean ± SD]	76.1 ± 22.2	75.3 ± 25.9	n.s
Troponin [pg/mL, median (IQR)]	3656.0 (1074.0–6792.0)	3282.5 (969.5–6055.8)	n.s
**Biomarkers—after 24 months (selection)**			
LDL-C [mmol/L, mean ± SD]	1.2 ± 0.7	2.1 ± 1.04	** < 0.01**
**Outcome data—after 24 months**			
MACE [*n* (%)]	4 (7.5)	6 (18.8)	n.s
**Echocardiographic parameters—baseline**			
LVEF [%, mean ± SD]	50.6 ± 9.7	51.1 ± 11.8	n.s
**Comorbidities—baseline**			
Hypertension [*n* (%)]	33 (62.3)	21 (65.6)	n.s
CKD [*n* (%)]	32 (60.4)	20 (62.5)	n.s
PAD [*n* (%)]	5 (9.4)	4 (12.5)	n.s
Diabetes [*n* (%)]	9 (16.9)	4 (12.5)	n.s

*p* < 0.05 = statistically significant, *SD* standard deviation, *BMI* body mass index, *PAD* peripheral artery disease, *CKD* chronic kidney disease, *CAD* coronary artery disease, *n.s.* not significant, *IQR* interquartile range, *eGFR* estimated glomerular filtration rate, *LDL-C* low-density lipoprotein cholesterol, *HDL* high-density lipoprotein, *LVEF* left ventricular ejection fraction, *GP* general practitioner, *MACE* major adverse cardiac events (nonfatal ischemic cardiovascular events, admission for heart failure, nonfatal stroke)

*Specialized lipid clinic vs. GP

### Factors affecting LDL-C goal attainment

**Fig. 1:**
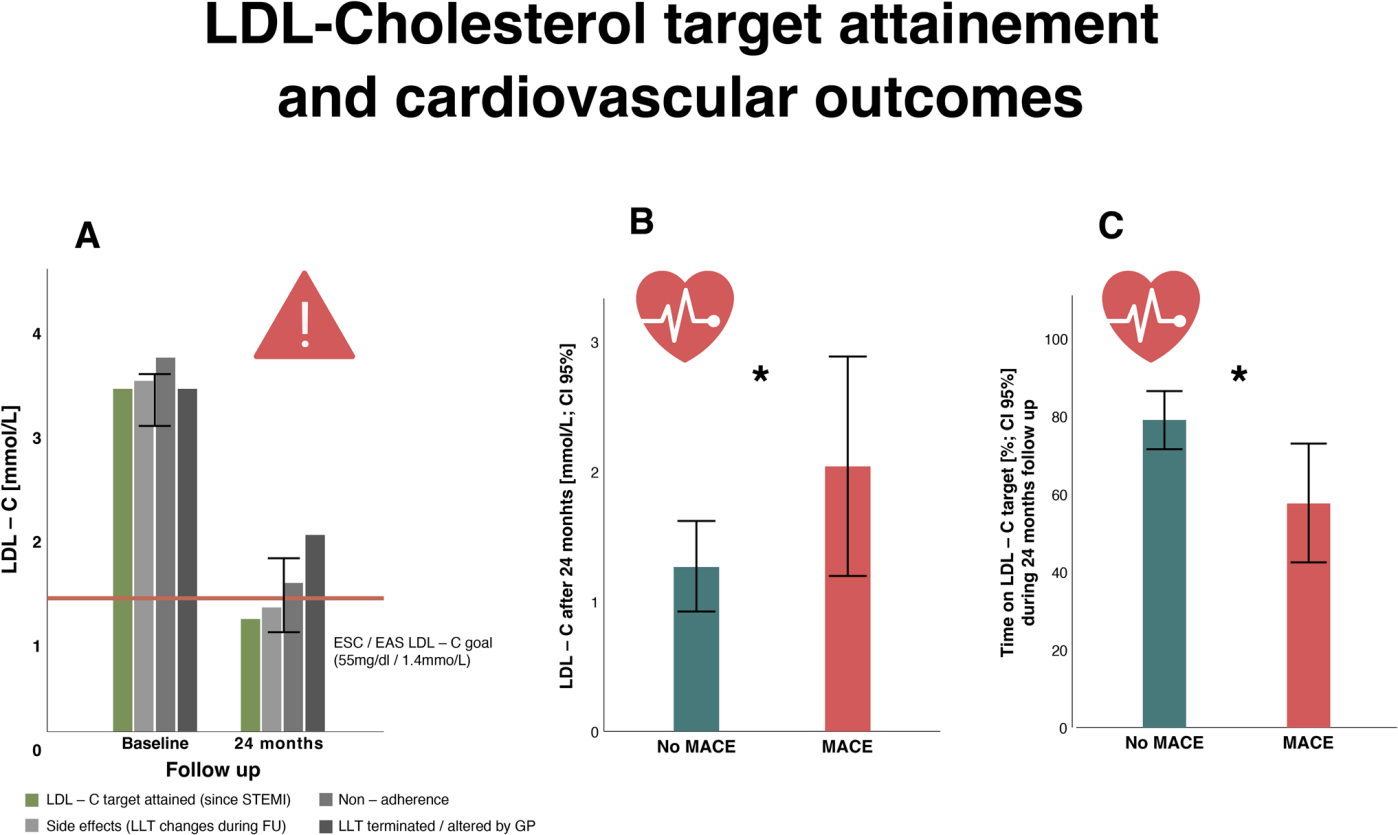
**A** LDL-C target attainment after 24 months stratified according to side effects, adherence, and alterations of LLT by GP. LLT = lipid-lowering therapy; CI = confidence interval; ESC = European Society of Cardiology; EAS = European Atherosclerosis Society; GP = general practitioner; STEMI = ST-elevation myocardial infarction; FU = follow up. *p < 0.05 for LDL-C goal attainment vs. LLT via GP. **B** Comparison between patients with and without MACE regarding LDL-C after 24 months. *p < 0.05. **C** Time on LDL­C target defined by ESC/EAS of 1.4 mmol/L and below after 24 months for no MACE vs. MACE. *p < 0.05. MACE = major adverse cardiac event

The primary reason for failing to achieve LDL-C targets was “deprescribing” of LLT by local GPs, which was more impactful than non-adherence to therapy (2.1 ± 1.04 mmol/L vs. 1.52 ± 0.53 mmol/L, *p* < 0.01), Fig. 1.

### MACE and LDL-C control

MACE which required a second hospital stay during the follow-up period occurred in 10 patients (12.3%). MACE patients were characterized by significantly shorter durations on LDL-C targets (58.1 ± 29.9% vs. 79.1 ± 28.1%; *p* = 0.048) and higher LDL-C levels at 24 months (2.04 ± 1.26 mmol/L vs. 1.27 ± 0.72 mmol/L; *p* < 0.01), Fig. 1. MACE rates were lower in the lipid clinic group (7.5% vs. 18.8%), though not statistically significant (Table 2). Higher vessel CAD and peripheral arterial disease (PAD) were significantly associated with MACE (Table 3). Although not statistically significant, comorbidities such as chronic kidney disease (CKD), elevated Lp(a), and reduced left ventricular ejection fraction (LVEF) were numerically more frequent in patients who experienced MACE, suggesting a possible association (Table 3).

**Table 3:** Characteristics of the study group: non-MACE vs. MACE patients

	Non-MACE patients	MACE patients	*p* Value*
	(*n* = 75 (88%))	(*n* = 10 (12%))	
**Demographics**			
Age [years, mean ± SD]	63.4 ± 12.9	70.3 ± 12.6	n.s
Male [*n* (%)]	65 (86.7)	8 (80.0)	n.s
Female [*n* (%)]	10 (13.3)	2 (20.0)	n.s
BMI [kg/m^2^, mean ± SD]	27.7 ± 4.1	28.4 ± 4.9	n.s
Smokers [*n* (%)]	29 (38.7)	4 (40.0)	n.s
Systolic blood pressure [mmHg, mean ± SD]	145.4 ± 27.6	146.6 ± 26.5	n.s
Diastolic blood pressure [mmHg, mean ± SD]	80.4 ± 18.0	80.1 ± 12.8	n.s
CAD [*n* (%)]	75 (100)	10 (100)	n.s
1 Vessel CAD [*n* (%)]	36 (48.0)	0 (0.0)	
2 Vessel CAD [*n* (%)]	19 (25.3)	4 (40.0)	** < 0.01**
3 Vessel CAD [*n* (%)]	20 (26.7)	6 (60.0)	
Number of stents [median (IQR)]	1 (1–2)	2 (1–3)	n.s
**Biomarkers**			
Total cholesterol [mmol/L, mean ± SD]	4.4 ± 1.4	4.2 ± 1.4	n.s
LDL-C [mmol/L, mean ± SD]	3.3 ± 1.2	3.1 ± 1.1	n.s
HDL-C [mmol/L, mean ± SD]	1.2 ± 0.4	1.1 ± 0.3	n.s
Triglycerides [mmol/L, mean ± SD]	1.5 ± 1.4	1.1 ± 0.5	n.s
Lipoprotein (a) [nmol/L, median (IQR)]	19.9 (19.9–59.0)	30.0 (19.9–177.8)	n.s
HbA1C [%, mean ± SD]	5.9 ± 0.7	6.1 ± 0.9	n.s
eGFR [mL/min, mean ± SD]	78.2 ± 21.0	62.4 ± 31.1	n.s
Troponin [pg/mL, median (IQR)]	1688.0 (916.0–5525.0)	4478.0 (1085.0–6832.0)	n.s
**Echocardiographic parameters**			
LVEF [%, mean ± SD]	51.6 ± 9.4	46.9 ± 14.5	n.s
**Comorbidities**			
Hypertension [*n* (%)]	48 (64.0)	6 (60.0)	n.s
CKD [*n* (%)]	44 (58.7)	8 (80.0)	n.s
PAD [*n* (%)]	6 (8.0)	3 (30.0)	** < 0.05**
Diabetes [*n* (%)]	12 (16.0)	1 (10.0)	n.s

*p* < 0.05 = statistically significant, *SD* standard deviation, *BMI* body mass index, *PAD* peripheral artery disease, *CKD* chronic kidney disease, *CAD* coronary artery disease, *n.s.* not significant, *IQR* interquartile range, *eGFR* estimated glomerular filtration rate, *LDL-C* low-density lipoprotein cholesterol, *HDL* high-density lipoprotein, *LVEF* left ventricular ejection fraction, *MACE* major adverse cardiac events (nonfatal ischemic cardiovascular events, admission for heart failure, nonfatal stroke)

*No MACE vs. MACE

### Additional clinical parameters

Baseline characteristics, including age, sex distribution, BMI, smoking status, blood pressure, and echocardiographic parameters such as left ventricular ejection fraction (LVEF), were similar between groups (Tables 1 and 3). Hypertension was more prevalent in the non-goal-attainment group (67.6% vs. 60.7%), though this difference was not statistically significant. Systolic blood pressure was significantly lower in the specialized lipid clinic group compared to the general practitioner (GP) group (140.4 ± 27.3 vs. 154.5 ± 26.5 mmHg, *p* = 0.024; Table 2). Other comorbidities, including diabetes, CKD, and PAD, showed no significant differences between groups, except in the MACE versus non-MACE subgroups, where CAD severity and PAD prevalence were higher in the MACE group. Biomarkers, including total cholesterol, high-density lipoprotein cholesterol (HDL-C), triglycerides, lipoprotein (a), HbA1c, and estimated glomerular filtration rate (eGFR), exhibited no significant intergroup differences.

## Discussion

“Jena auf Ziel” has demonstrated that stringent ESC/EAS LDL-C target attainment by early combination of a high-intensity statin with the cholesterol absorption inhibitor ezetimibe and escalation with bempedoic acid and PSCK9-I in patients with ST-elevation myocardial infarction (STEMI) is feasible, effective, and safe over a follow-up period of 12 months. The 2-year follow-up results presented in this analysis, however, are sobering as a substantial proportion of patients have failed to stay on ESC/EAS LDL-C targets 2 years after the index event. Patients managed in the outpatient lipid clinic of the University Hospital Jena had significantly better LDL-C goal attainment over time compared to patients treated by their local GPs. “Deprescribing” of previously implemented LLT was the main reason for LDL-C target failure after 24 months and was associated with higher LDL-C levels and major adverse cardiovascular events (MACEs).

The results of the current analysis highlight a significant structural gap in the treatment landscape of high-risk ASCVD patients in Germany.

Regarding the discrepancies in target achievement rates between the outpatient clinic of the University hospital in Jena and treatment by local GPs, it is essential to consider that disparate targets are applicable to these settings. While the ESC/EAS guidelines [5], which are relevant for cardiologist, recommend LDL-C reduction of ≥ 50% from baseline and a LDL-C target of < 55 mg/dL in very high-risk patients, the Nationale Versorgunsleitline (NVL) [13], which is followed by most GPs, recommends a “fire and forget” strategy and less aggressive lipid lowering with higher LDL-C targets (70 mg/dL).

In addition to discrepancies in guideline recommendations, current reimbursement restrictions for PCSK9 modifying therapies in Germany may contribute to these divergent results. The current findings indicate that bempedoic acid (BA) as well as PSCK9-I, which were initiated during the first 12 months of follow-up in the outpatient lipid clinic of the Jena University Hospital, were “deprescribed” in the GP setting during the second year of follow-up. In Germany, PCSK9-I must be initiated by specialists, including those in the fields of cardiology, nephrology, endocrinology, diabetology, and angiology, as well as by specialists in lipid outpatient clinics. Subsequently, a continuous prescription is also feasible in the GP setting, but, due to possible financial restrictions, is often not translated in daily clinical practice.

It is unfortunate that treatment options that are not generically available, such as BA and PCSK9-I, are not being utilized to their fullest potential, given the effectiveness of these options [8, 9]. Moreover, it is concerning that in high-risk patients after myocardial infarction, well-tolerated drugs are deprescribed without medical indications which leads to higher LDL-C levels and, most importantly, higher incidence of MACE.

The discrepancies observed earlier in LipidSnaphot and the current analysis of the “Jena auf Ziel” cohort between the OBC and the GP setting prompt the question of whether the NVL guidelines should be updated to meet best practice standards with more stringent LDL targets. This is all the more important since most patients with elevated levels of cholesterol are treated by GPs and not by cardiologists [15]. Therefore, enhanced cross-sectional networking could potentially improve patient care. Given the efficacy of available LLT, the rates of target achievement and adherence in clinical practice are concerning.

Our results parallel findings from the multinational SANTORINI study, which reported that only 20.1% of high- and very high-risk patients achieved guideline-recommended LDL-C levels in Europe, largely due to underutilization of combination LLT and inconsistent adherence to guidelines [8]. Koenig and colleagues reported only recently a retrospective analysis from German claims data that there are high discontinuation rates of LLT, with statins having the lowest long-term persistence [16]. These findings underscore the challenges of long-term adherence to LLT and the need for improved strategies to optimize LDL-C control [17].

Our study identified a significant association between LDL-C levels and MACE incidence, with patients experiencing MACE having higher LDL-C levels and shorter durations at target LDL-C. This aligns with recent findings from SWEDEHEART, which demonstrated that patients with sustained reductions in non-HDL-C had the lowest risk of MACE [10, 12]. The importance of sustained LDL-C reduction is further supported by evidence indicating that higher LDL-C exposure over time contributes to atherosclerotic cardiovascular disease [18].

Our findings reinforce the necessity for systematic approaches to LDL-C management, including standardized treatment algorithms and improved adherence strategies. Given that “deprescribing” by GPs was a significant barrier to LDL-C goal attainment over time, targeted education and guideline reinforcement for primary care providers may be beneficial. Moreover, the result of the current analysis are a clear call to guideline harmonization in Germany. Differing treatment algorithms and LDL-C target recommendations by medical societies is a major driver for confusion. Furthermore, expanding access to lipid clinics and utilizing telemedicine-based interventions could improve long-term adherence to LLT. Future research should explore the implementation of structured LDL-C management programs and assess their impact on long-term cardiovascular outcomes.

## Conclusion

The 2-year follow-up of “Jena auf Ziel” suggests that adherence to evidence-based guidelines and structured follow-up programs may improve LDL-C goal attainment and reduce cardiovascular outcomes. Addressing barriers such as “deprescribing” by local GPs and patient adherence remains a critical priority for optimizing secondary prevention strategies in very high-risk populations such as in patients after STEMI.

## Limitations

This is a single-center and retrospective study with associated limitations.

## Data Availability

The data presented in this study is not publicly available due to local legal restrictions on data safety.
